# Thin-Ideal Internalisation and Weight Bias Internalisation as Predictors of Eating Pathology: The Moderating Role of Self-Compassion

**DOI:** 10.3390/ijerph22081278

**Published:** 2025-08-15

**Authors:** Gillian Montague, Taban Eidipour, Sharon L. Grant

**Affiliations:** 1 Department of Psychological Sciences, School of Health Sciences, Swinburne University of Technology, Hawthorn, VIC 3122, Australia; 2 Clinical Psychology Team, Peter MacCallum Cancer Centre, 305 Grattan Street, Melbourne, VIC 3000, Australia; 3 Specialist Medical Inpatient Team, Sunshine Hospital, Western Health, 176 Furlong Road, St Albans, VIC 3021, Australia

**Keywords:** disordered eating, thin-ideal internalisation, weight bias internalisation, self-compassion

## Abstract

The internalisation of sociocultural ideals and beliefs about weight and shape has long been recognised as an important predictor of disordered eating. However, thin-ideal internalisation and weight bias internalisation (WBI) are generally examined separately in the literature and within sociocultural models of eating disorders. Additionally, self-compassion has been identified as a protective factor against disordered eating, but its role in mitigating the impact of the internalisation of these sociocultural ideals and beliefs has not been investigated. The current study aimed to investigate (1) the unique contribution of thin-ideal internalisation and WBI in predicting eating disorder cognitions and behaviours and (2) the role of self-compassion in moderating the relationship between thin-ideal/WBI and eating pathology. Four hundred and seventy-five (475) women completed an anonymous online survey. The results indicated that both thin-ideal internalisation and WBI uniquely contributed to the prediction of eating pathology after controlling for body mass index. Self-compassion buffered the effect of thin-ideal internalisation on restraint and the effect of WBI on eating concern. The results support consideration of both thin-ideal internalisation and WBI in sociocultural models of eating disorders and as targets for eating disorder interventions, particularly those based on self-compassion therapy.

## 1. Introduction

The global prevalence of eating disorders has continued to rise in recent decades, more than doubling from 3.5% in 2000 to 7.8% in 2018 [1]. In addition to those who meet diagnostic criteria for eating disorders, it is likely that an even larger proportion of individuals experience sub-threshold eating pathology [2]. Thus, developing a further understanding of the aetiology of disordered eating and effective treatment approaches remains a key focus for research.

A significant body of research on the factors that contribute to the development and maintenance of disordered eating, particularly among women, has highlighted the influential role of sociocultural ideals and beliefs about body weight and shape [3,4,5]. Western societies have long promoted the ‘thin ideal’, equating a slim physique with feminine beauty and virtue. This ideal is reinforced through sociocultural influences such as the media (e.g., advertising, magazines, television), peers, and family members [6]. Corresponding to the thin ideal is the stigmatisation of overweight and obesity, through the negative stereotyping of individuals with larger bodies as ‘lazy’ and ‘undisciplined’, with the implication that they are personally responsible for their weight [6,7,8]. Research indicates that these stigmatising beliefs manifest in the community, with heavier individuals being teased, treated unfairly, or discriminated against based on their weight [9]. Individuals may experience weight stigma from a range of sources, including classmates, colleagues, doctors, family members, and friends, with individuals of higher body mass index (BMI) reporting more frequent weight stigmatising experiences [9,10].

Research has demonstrated that the internalisation of the thin ideal and weight stigma contributes to the development and maintenance of disordered eating [3,4,5]. Thin-ideal internalisation can be defined as the “extent to which an individual cognitively ‘buys into’ socially defined ideals of attractiveness [associated with thinness] and engages in behaviours designed to produce an approximate of these ideals” [5] (p. 181).

Sociocultural models of eating disorders predominantly focus on the role of thin-ideal internalisation in eating disorder development and maintenance [6,11]. A leading sociocultural model of eating disorders, the tripartite influence model of body image and eating disturbance [6], suggests that individuals experience sociocultural pressure to be thin, which is reinforced by sources including the media, family, and peers. Pressure to be thin leads to thin-ideal internalisation and appearance comparison with others, which, in turn, leads to body dissatisfaction and disordered eating. The dual pathway model of bulimia nervosa [11] proposes a similar model, suggesting that pressure to be thin leads to thin-ideal internalisation and, in turn, body dissatisfaction. This then leads to dietary restraint and negative affect, ultimately resulting in bulimic pathology.

These models have garnered significant support within the literature. Research has demonstrated that exposure to the thin ideal in the media is positively associated with thin-ideal internalisation and the frequency of cognitions and behaviours related to anorexia and bulimia [12]. Findings have also highlighted negative outcomes associated with thin-ideal internalisation, including increased body dissatisfaction, negative affect, dieting, and bulimic symptoms [5,13]. Additionally, longitudinal research has shown that thin-ideal internalisation is predictive of future onset of both sub-threshold and threshold bulimia [14].

Based on evidence for sociocultural models of disordered eating, interventions have been developed that aim to reduce disordered eating and prevent eating disorder onset by targeting thin-ideal internalisation. One such program with an extensive evidence base is the cognitive dissonance-based intervention, the Body Project [15,16]. This intervention involves participants generating critiques of the thin ideal through verbal, written, and behavioural tasks; for example, identifying costs of pursuing the thin ideal and engaging in role-plays that involve convincing others not to pursue the ideal [16]. Findings reveal that the Body Project leads to significant reductions in thin-ideal internalisation, body dissatisfaction, negative affect, dieting, and eating disorder symptoms [15]. Additionally, these effects are greater than those produced by alternative interventions, such as educational videos or the Healthy Weight program [15,17]. Although this intervention was initially designed for use with women, further research has demonstrated that it can be modified to target eating disorder risk factors among men, including more specific male groups, such as male athletes and gay men [18]. These modified versions of the Body Project have led to reductions in body dissatisfaction, including dissatisfaction with muscularity, muscle dysmorphia, drive for muscularity, and dissatisfaction with body fat [18].

Weight bias internalisation (WBI) is defined as the awareness, agreement, and application of weight stigma to the self, resulting in devaluation of oneself based on body weight [19]. As explained by Harris et al. [20], thin-ideal internalisation and WBI represent the internalisation of different societal shape and weight ideals, emphasising the value of thinness and the devaluation of larger body size, respectively. The authors investigated the extent to which the two constructs are distinct, arguing that thin idealisation might overlap with larger body size devaluation or, alternatively, that these beliefs may be separable. They reported that thin-ideal internalisation and WBI contribute uniquely to the prediction of body dissatisfaction and that the relationship between the two constructs did not differ based on body size. However, only WBI moderately correlated with body size. The authors concluded that the thin-ideal may be internalised regardless of body size, whereas individuals of larger body size may be more prone to WBI because they more strongly associate their identity with sociocultural beliefs about overweight. Indeed, other research has found that WBI is positively correlated with BMI, such that individuals with higher BMIs experience greater levels of WBI [4].

While most research into WBI focuses on individuals with overweight or obesity, findings suggest that WBI is positively associated with binge eating and purging behaviours among individuals of varying BMIs [3,4]. Individuals with higher WBI experience greater levels of disordered eating, depression and anxiety, poorer self-esteem and body image, and lower motivation/self-efficacy to participate in health-promoting behaviour, e.g., physical exercise [19]. A study by Romano et al. [3] suggested that the mechanisms behind the association between WBI and disordered eating may be like those proposed by the tripartite influence model. The findings provided support for a model wherein weight stigmatising experiences predict WBI, which, in turn, predicts body dissatisfaction, leading to disordered eating. Therefore, both models suggest that both sociocultural pressures to be thin (i.e., to meet body ideals), or not to be fat (i.e., to avoid weight stigma), lead to the internalisation of societal ideals/beliefs around body weight and shape, which then lead to body dissatisfaction and, subsequently, eating pathology [3,6].

To date, thin-ideal internalisation and WBI have largely been examined separately within the literature, with a paucity of research examining the unique and combined effects of these two factors in disordered eating. While both constructs relate to the internalisation of societal ideals or beliefs around body weight and shape, they are theoretically distinct [20]. Supporting this notion are research findings demonstrating that thin-ideal internalisation and WBI differ in their relationships to related constructs. For example, WBI is positively correlated with BMI and is predominantly associated with binge eating, whereas thin-ideal internalisation does not appear to be correlated with BMI and is predominantly associated with dieting and bulimic pathology, including purging behaviours [4,14,20,21]. A recent study by Nutter et al. [22] suggested that the relationship between thin-ideal internationalisation and anti-fat attitudes, a form of weight stigma, is mediated by appearance comparisons. While Harris et al. [20] examined the relationship between both constructs and body dissatisfaction across different BMI categories, they did not examine moderators of the relationship between thin-ideal internalisation or WBI and eating pathology, highlighting a gap in the literature. Further research is required to better understand the unique contribution of each construct to the prediction of eating disorder cognitions and behaviours, including moderating factors, and how targeting both forms of internalisation may enhance the effectiveness of intervention strategies [20].

As noted by Morton et al. [23], the internalisation of weight-related sociocultural ideals/beliefs is considered extremely common within the population; thus, it is beneficial to understand protective factors which may prevent individuals who have internalised these views from developing disordered eating. One such protective factor is self-compassion [24,25,26], which refers to an individual’s ability to direct compassion inwardly and treat themselves with care and support in response to distress and self-perceived flaws [27,28]. Self-compassion consists of three facets that combine and interact to influence compassionate/uncompassionate responses: (1) kindness (e.g., soothing and comforting oneself) versus self-judgment (e.g., blaming and judging oneself), (2) common humanity (e.g., recognising that imperfection is shared by all humanity) versus isolation (feeling detached and isolated during difficult times), and (3) mindfulness (acknowledging distress without amplifying or suppressing it) versus overidentification (becoming overwhelmed and consumed by distress) [27,28]. Research suggests that individuals who exhibit greater self-compassion experience higher levels of overall psychological wellbeing, including lower psychopathology, such as depression, anxiety, and stress [29], and higher psychological strengths, including happiness and optimism [30].

Within the eating disorder literature, Tylka and Huellemann [31] discussed the relevance of self-compassion for body-related distress and perceived body flaws. They framed thin-ideal internalisation and weight bias internalisation in terms of body image threat. In this context, kindness (versus self-judgement) translates into nurturing rather than judging the body; common humanity (versus isolation) reflects understanding that societal appearance ideals can amplify body image concerns; and mindfulness manifests as thinking about the body in a more accepting and balanced way, e.g., body appreciation rather than self-objectification. Self-compassion has been negatively associated with body dissatisfaction and disordered eating patterns [24,25,26,32,33]. It has also been negatively associated with shape and weight concern and body preoccupation [26]. A systematic review of 28 studies showed that self-compassion was consistently linked with better body image and lower eating pathology through simple mediation and serial mediation pathways involving maladaptive body image and eating disorder risk factors, with some evidence of buffering effects [24]. A comparative study of university students and patients with diagnosed eating disorders reported significantly lower levels of self-compassion and greater fear of self-compassion in the patient group [25]. Longitudinal research suggests that low self-compassion may be predictive of eating disorder risk, with results of one study [32] indicating that higher levels of self-compassion at baseline predicted a lower likelihood of experiencing the onset of eating disorder symptoms at an eight-month follow-up.

Based on relationships between self-compassion and numerous factors related to disordered eating, researchers have begun investigating the efficacy of self-compassion for reducing disordered eating and associated risk factors. Linardon et al. [33] examined self-compassion as a moderator of the relationship between shape/weight overvaluation and eating disorder psychopathology, psychosocial impairment, and psychological distress in men and women. They reasoned that self-compassion may be a protective factor given its role as an adaptive affect regulation strategy. Their findings suggested that overvaluation of shape and weight was associated (or associated more strongly) with the outcomes for individuals with low levels of self-compassion. Other research [34] has shown that self-compassion is associated with lower WBI, which is, in turn, associated with lower emotional and restrained eating.

When considered within sociocultural models of eating disorders, research suggests that self-compassion may function as a protective factor against societal pressures related to disordered eating [34,35,36]. The findings of a study by Tylka et al. [36] indicated that individuals with higher levels of self-compassion perceived less sociocultural pressure to be thin. Self-compassion also acted as a buffer in the relationship between pressure to be thin (media) and thin-ideal internalisation and disordered eating, such that when self-compassion was low, pressure to be thin was more strongly related to both thin-ideal internalisation and disordered eating [36]. However, self-compassion has not been examined as a moderator of the effect of WBI, or thin-ideal internationalisation, on eating pathology in previous studies. A meta-analysis by Turk and Waller [37] revealed that self-compassion-focused interventions were effective in reducing body image concerns and disordered eating. Such interventions may also be effective in reducing risk factors for eating disorder development, such as the internalisation of sociocultural ideals. Gobin et al. [38] demonstrated that implementation of a brief self-compassion intervention prior to exposure to thin-ideal images on social media prevented subsequent increases in weight and appearance dissatisfaction that were experienced by individuals who did not receive the intervention. Self-compassion interventions have also demonstrated effectiveness in reducing WBI, with a recent study by Hopkins [39] finding that a 28-day digital, mindful self-compassion intervention led to significant reductions in WBI when compared to a waitlist control. While research has demonstrated that self-compassion may serve as a protective factor against the internalisation of sociocultural ideals and beliefs relating to weight and shape, there is a need to investigate whether self-compassion moderates the relationship between thin-ideal internalisation/WBI and disordered eating.

**Figure 1:**
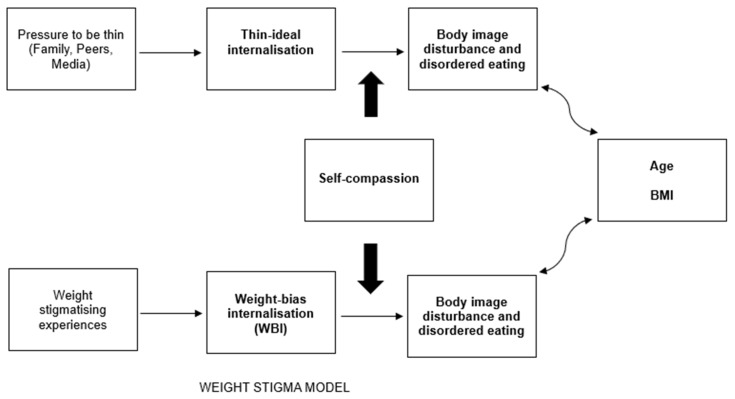
Self-compassion as a moderator of the relationship between internalisation and body image disturbance and disordered eating in sociocultural models of eating pathology. Note. Light, unidirectional arrows depict mediated pathways. Heavy arrows depict moderation. Bidirectional arrows depict reciprocal effects. The bolded relationships in Figure 1 are tested in this study.

The aims of the present study are twofold. Firstly, the current study aims to extend the literature regarding sociocultural factors associated with disordered eating by investigating the unique contributions of thin-ideal internalisation and WBI in predicting eating disorder cognitions and behaviours. Secondly, this study aims to investigate the potential protective role of self-compassion in moderating the relationship between (a) thin-ideal internalisation and (b) WBI and disordered eating. Figure 1 depicts the integration of self-compassion as a moderator in sociocultural models of eating disorders. Based on the previous findings by Harris [20], it is hypothesised that thin-ideal internalisation and WBI independently predict eating pathology. Due to the current lack of research related to the moderating role of self-compassion in these relationships, the moderated effects analysis was considered exploratory, and no a priori hypotheses were generated.

## 2. Materials and Methods

Adults who identified as female were eligible to participate and were recruited via a research experience program at an Australian university and the researchers’ social media networks as part of a larger two-part study investigating self-compassion as a mediator of the relationship between body-positive media and body-image-related outcomes. An eligibility criterion for only part two of the larger study was that the participants had not previously viewed the body positivity documentary film, Embrace (see Embrace: The Documentary (2016)—IMDb). The study compared the effect of informational, narrative, and persuasive appeal segments from this body positivity documentary on pre-test and post-test changes in self-compassion, thin-ideal internalisation, and WBI (see Measures below) as well as self-esteem (not reported here). The current study utilises data from part one of the larger study only.

Four hundred and eighty (480) women participated in the first survey. Two univariate outliers for BMI were deleted, and three multivariate outliers were deleted, reducing the final sample to 475. Age ranged from 18 to 67 years (*M* = 31.48, *SD* = 10.62). Most participants identified as Australian/Caucasian (67%), and the remaining participants identified as European (7%), South Asian (7%), Asian (6%), African (3%), Middle Eastern (3%), Pacific Islander (1%), or multi-ethnicity (4%). Body mass index (BMI) ranged from 14.54 to 66.6, with 46% of the participants falling within the healthy weight range, 4% within the underweight range, and 50% falling within the pre-obesity to obesity range [40]. The average Eating Disorder Examination-Questionnaire global score for the sample (*M* = 3.06, *SD* = 1.23) was above the clinical cut-off of greater than or equal to 2.5 [41]. The survey measures are described below.

The Modified Weight Bias Internalisation Scale (WBIS-M) [42] is an 11-item scale that measures the extent to which individuals evaluate themselves based on negative, weight-based stereotypes. Derived from the Weight Bias Internalisation Scale [43], the WBIS-M is suitable for individuals from a range of BMI categories. Responses are based on a 7-point Likert scale from 1 (strongly disagree) to 7 (strongly agree), with higher overall scores (averaged) indicating greater WBI.

The Internalisation Scale of the Sociocultural Attitudes toward Appearance Questionnaire 4—Revised (SATAQ-4R) [44] contains five questions that measure the internalisation of the thin ideal using a five-point Likert scale from 1 (definitely disagree) to 5 (definitely agree), with higher scores (averaged) indicating greater levels of internalisation.

The State Self-Compassion Scale—Long Form (SSCS-L) [45] is an 18-item scale that assesses self-kindness, self-judgment, common humanity, isolation, mindfulness, and over-identification in the moment on a Likert scale from 1 (not at all true for me) to 5 (very true for me). Higher overall scores (averaged) indicate greater levels of state self-compassion.

The Eating Disorder Examination—Questionnaire (EDE-Q) [46] is a 28-item measure of eating disorder cognitions and behaviours in the preceding 28 days. The measure consists of four subscales: eating concern, restraint, shape concern, and weight concern. A global score is obtained by averaging the respondents’ subscale scores. Higher scores indicate greater levels of eating pathology, with a global score of 2.5 or greater indicating eating pathology [39].

The shape concern (concern about the body’s appearance) and weight concern (concern about the body’s measured weight) subscales are often utilised to assess body dissatisfaction. Questions have been raised regarding conceptual overlap between EDE-Q items that measure weight concern and WBIS items [47,48] (e.g., EDE-Q items ‘Have you had a strong desire to lose weight?’, ‘Has your weight influenced how you think about (judge) yourself as a person?’, and ‘How dissatisfied have you been with your weight?’, and WBIS items ‘I wish I could drastically change my weight’, ‘My weight is a major way that I judge my value as a person’, and ‘I am ok being the weight that I am’). As such, weight concern was not investigated as an outcome variable in the current study.

The participants were asked to provide their age, gender (male, female, non-binary/third gender, prefer not say), ethnicity, and current weight (kilograms [kg]) and height (metres [m]) for calculation of BMI (weight [kg]/(height [m]^2^).

We note that the appropriateness of using BMI has been debated. For example, Byker Shanks et al. [49] presented arguments for and against its use as an individual-level and/or population-level health measure for adults. They argued that while BMI is a cost-effective and feasible measure, several issues call use of the metric into question, including its historical association with eugenic ideology (an ‘ideal’ body); standardisation of weight categories to White European, mainly male populations; limitations for understanding body composition and health risk; and inconsistent findings regarding racial, ethnic, and sex differences.

This study was advertised online via Sona Systems (used to host the research experience program [REP]) and social media. Prospective participants received a link to the online survey, which they could complete voluntarily via Qualtrics at their convenience. After reading the consent information statement, the participants completed screening questions for the eligibility criteria (identify as female, aged 18 years or older), with those who did not meet the criteria being directed out of the survey immediately. The remaining participants completed the measures, as ordered above, and one additional questionnaire for the larger study. Consent was implied by submission of a completed survey. As part of the larger study, eligible participants were required to complete a second survey three days later. Following completion of both surveys, REP participants were awarded course credit in exchange for their participation. There were no other incentives.

## 3. Results

### 3.1. Overview of Analysis

Moderated regression analysis [50] was used to determine (1) the unique contribution of thin-ideal internalisation and WBI in the prediction of eating pathology, (2) the interaction of thin-ideal internalisation and self-compassion in the prediction of eating pathology, and (3) the interaction of WBI and self-compassion in the prediction of eating pathology. In moderated regression analysis, the interaction between two (or more) predictors, ‘X’ and ‘Z’, is represented by their multiplicative interaction term or cross product, i.e., ‘XZ’. Since the interaction effect is the part of the cross product that is independent of the main effects, the interaction term is entered hierarchically, after the entry of the predictors involved in the interaction. Accordingly, the main effects of the predictors are partialled or removed from the interaction effect. The moderated effect model is supported if there is a significant increment in *R*^2^ when the interaction term is added to the regression equation. That is, if the interaction effect produces a significant increment in the total variance explained over and above the main effect (s) of the component predictors. If the regression coefficient for a given interaction term is significant, the next step is to plot the regression lines to examine the simple slope of Y (outcome) on X (predictor) at each value of Z (moderator). If the lines for the low and high values of Z are parallel, then there is no interaction effect, i.e., the regression of Y on X does not vary as a function of Z. If the lines are divergent, then an interaction effect is present. A simple slope analysis is then conducted to interpret the interaction effect, specifically whether the simple slope of Y on X significantly differs from zero at each value of Z (i.e., low, high). Total scores for thin-ideal internalisation, WBI, and self-compassion were mean-centred prior to analysis. Interaction terms were computed based on these mean-centred variables to prevent multicollinearity between the main effects and interaction effects [50]. Separate moderated regression analyses were conducted with restraint, eating concern, and shape concern as outcomes. The general structure of the regression equation was the same for all models; however, where applicable, age and BMI were controlled and entered at the first step, prior to entry of other predictors.

### 3.2. Data Screening

All analyses were conducted using IBM SPSS Statistics 29. First, the data were examined for missing values. One case was missing a single value of the self-compassion measure, which was replaced using mean imputation. There were no other missing data. BMI and eating concern were positively skewed. No transformation was applied to BMI to preserve the original metric of the variable; however, a square root transformation was effectively applied to eating concern. All other variables were normally distributed. Table 1 presents the raw means, standard deviations, bivariate correlations, and Cronbach’s alpha (where applicable) for key study variables and potential covariates, i.e., age and BMI. All scales displayed good internal consistency. Variables that failed to correlate significantly with the eating pathology outcomes at the bivariate level were excluded from subsequent multiple regression models.

### 3.3. Self-Compassion as a Moderator of the Relationship Between Thin-Ideal Internalisation and WBI and Eating Pathology

#### 3.3.1. Restraint

The regression model exploring predictors of restraint included BMI in step one, thin-ideal internalisation, WBI, and self-compassion in step two, and the two interaction terms (thin-ideal internalisation × self-compassion, WBI × self-compassion) in step three (see Table 2). *R* was significantly different from zero at the end of each step. After step three, with all predictors included in the regression model, *R* = 0.50, *F* (6, 468) = 26.27, and *p* < 0.001. Thin-ideal internalisation and WBI were significant, positive predictors of restraint, and the interaction between thin-ideal internalisation and self-compassion was significant. The final model accounted for 24% of the variance in restraint. Thin-ideal internalisation was the strongest predictor of restraint.

**Figure 2:**
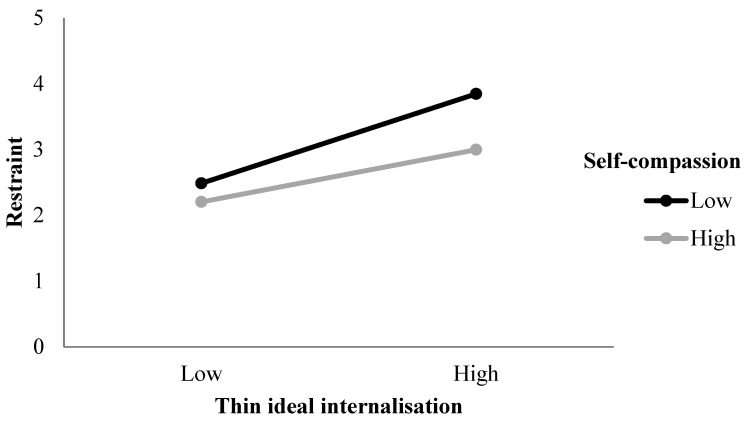
Two-way interaction between thin-ideal internalisation and self-compassion in the prediction of restraint.

A plot of the interaction between thin-ideal internalisation and self-compassion in the prediction of restraint (Figure 2) showed that at low values of thin-ideal internalisation, there is little difference in the effect of low versus high values of self-compassion on restraint. However, at high values of thin-ideal internalisation, low values of self-compassion exacerbate its effect on restraint. Restraint is highest when thin-ideal internalisation is high and self-compassion is low. Simple slope analysis revealed that the slope of the regression line for low self-compassion was significantly different from zero, *t* (195) = 3.44, *p* = 0.001.

#### 3.3.2. Eating Concern

The regression model exploring the predictors of eating concern included age and BMI in step one, thin-ideal internalisation, WBI and self-compassion in step two, and the two interaction terms in step three (see Table 3). *R* was significantly different from zero at the end of each step. The addition of the interaction terms in step 3 produced a significant change in *R*^2^ (*p* = 0.005). After step three, with all predictors included, *R* = 0.72, *F* (7, 467) = 73.62, and *p* < 0.001. Both thin-ideal internalisation and WBI contributed uniquely to the prediction and were significant, positive predictors of eating concern. In addition, self-compassion was a significant negative predictor of eating concern, and the interaction between WBI and self-compassion was significant. Overall, the final model explained 52% of the variance in eating concern. WBI was the strongest predictor of eating concern.

**Figure 3:**
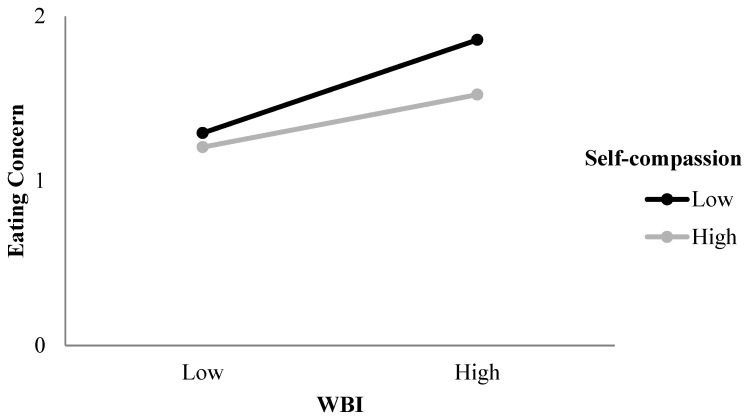
Two-way interaction between WBI and self-compassion in the prediction of eating concern.

A plot of the interaction between WBI and self-compassion in the prediction of eating concern (Figure 3) indicated that at low values of WBI, there is little difference in the effect of low versus high values of self-compassion on eating concern. However, at high values of WBI, low values of self-compassion exacerbate its effect on eating concern. Thus, eating concern is highest when WBI is high and self-compassion is low. Simple slope analysis revealed that the slope of the regression line for low self-compassion was significantly different from zero, *t* (195) = 3.44, *p* = 0.001.

#### 3.3.3. Shape Concern

The regression model for shape concern included age and BMI in step one, thin-ideal internalisation, WBI, and self-compassion in step two, and the two interaction terms in step three (see Table 4). *R* was significantly different from zero at the end of each step. After step three, with all predictors included, *R* = 0.83, *F* (7, 467) = 147.41, and *p* < 0.001. The addition of the interaction terms in step three did not produce a significant change in *R*^2^, and neither interaction term reached significance at the *p* < 0.05 level, indicating that there was no interaction between thin-ideal internalisation and self-compassion or WBI and self-compassion in the prediction of shape concern. In the final model, age, WBI, and thin-ideal internalisation were significant positive predictors of shape concern, each contributing uniquely to the prediction. In addition, self-compassion was a significant negative predictor of shape concern. WBI was the strongest predictor of shape concern. Overall, the final model accounted for 69% of the variance in shape concern.

## 4. Discussion

It has long been posited that thin-ideal internalisation and WBI are important contributing factors to the development and maintenance of disordered eating [3,4,5]; however, these constructs have generally been examined separately within the literature. The current study sought to examine the unique contribution of both constructs in predicting restraint, eating concern, and shape concern to build a more comprehensive understanding of how the internalisation of different sociocultural ideals and beliefs about weight and shape affects eating pathology. This study also aimed to explore the potential role of self-compassion in moderating the relationship between both forms of internalisation and eating disorder cognitions and behaviours. The findings of the current study suggest that the inclusion of both thin-ideal internalisation and WBI in sociocultural models of eating pathology may lead to the development of more effective intervention strategies for reducing disordered eating. Additionally, the findings support previous research [24,25,32] by revealing that lower self-compassion may exacerbate the effect of eating disorder risk factors on some outcomes. These findings lend further support to the utility of self-compassion therapy-based interventions for reducing disordered eating [37].

In line with previous studies (see, e.g., [20,22]), the findings support the consideration of thin-ideal internalisation and WBI as distinct but related theoretical constructs. Thin-ideal internalisation and WBI were moderately correlated and differed in their associations with BMI and restraint, eating concern, and shape concern. Consistent with previous studies (see, e.g., [20]), BMI and thin-ideal internalisation were not correlated, while BMI and WBI were positively correlated. This is likely due to the demonstrated link between BMI and weight stigmatising experiences; individuals with higher BMI report more weight stigmatising experiences, which, in turn, lead to higher internalisation of anti-fat attitudes and beliefs [3,51].

The current findings extend upon recent research indicating that thin-ideal internalisation and WBI uniquely predict body dissatisfaction [20] by demonstrating that both forms of internalisation uniquely predict both shape concern and disordered eating behaviours (restraint, eating concern). While thin-ideal internalisation was the strongest predictor of restraint, WBI was the strongest predictor of both eating concern and shape concern. In addition, the findings demonstrate that the effects of internalisation on disordered eating behaviour are moderated by self-compassion. Low self-compassion exacerbated the effect of thin-ideal internalisation on restraint and the effect of WBI on eating concern. These findings suggest that the inclusion of both thin-ideal internalisation and WBI in sociocultural models of body image and eating disturbance in future research is likely to improve our conceptual understanding of eating disorder cognitions and behaviours and inform the development of more effective prevention and intervention programs.

The tripartite influence model [6] posits that sociocultural pressure to be thin results in thin-ideal internalisation, leading to body dissatisfaction and disordered eating. Expansion of this model to incorporate WBI includes consideration of sociocultural factors related to the development of WBI, including anti-fat attitudes, negative obesity stereotypes, and weight-related discrimination, which may come from a range of sources [3,51]. Therefore, the model could be extended to include both sociocultural pressures to be thin and social stigmatisation of overweight/obesity as antecedents of thin-ideal internalisation and/or WBI, which then contribute to body dissatisfaction [see 20] and disordered eating. Further research is needed to examine whether pressure to be thin is positively related to WBI and whether weight stigmatising experiences are positively related to thin-ideal internalisation, so that sociocultural models of eating disorders can be expanded and integrated to include pathways between constructs from both the thin-ideal internalisation and weight stigma literature.

As expected, based on past research [34,36], self-compassion was negatively correlated with both thin-ideal internalisation and WBI, such that individuals with higher self-compassion reported lower thin-ideal and weight bias internalisation. Congruent with previous findings [24,25,32], self-compassion was also a negative predictor of eating disorder cognitions. However, self-compassion did not predict restraint after accounting for thin-ideal internalisation and WBI. It may be that fear of self-compassion (e.g., weakness, loss of control) rather than self-compassion per se is a stronger predictor of restrained eating (see, e.g., [52]).

In terms of the moderating role of self-compassion in the relationship between the internalisation constructs and restraint, eating concern, and shape concern, the results differed across the outcome variables. Self-compassion moderated the effect of thin-ideal internalisation, but not WBI, on restraint. In contrast, self-compassion moderated the effect of WBI, but not thin-ideal internalisation, on eating concern. Notably, thin-ideal internalisation and WBI were the strongest predictors of restraint and eating concern, respectively. These stronger predictors may be more sensitive to variability in vulnerability/resistance factors. For individuals with lower levels of self-compassion (i.e., critical, judgemental, and emotionally unforgiving), there was a stronger association between thin-ideal internalisation and restraint and weight bias and eating concern. In contrast, those with higher levels of self-compassion appeared to be protected from the effects of these sociocultural influences. This is consistent with previous research citing the protective role of self-compassion in eating pathology [24,25,32] and reinforces the utility of strengthening self-compassion within eating disorder interventions [37].

Finally, self-compassion did not moderate the relationship between thin-ideal internalisation or WBI and shape concern; however, the main effects explained a higher proportion of the total variance for this outcome. While previous research has demonstrated that self-compassion is negatively correlated with thin-ideal internalisation and WBI [34,36], the current results suggest that when individuals have already internalised these body ideals/beliefs, self-compassion does not buffer their effects on shape concern, even if it buffers their effects on related eating pathology. Notably, of the three EDE-Q subscales examined, shape concern showed the highest average score, suggesting that it was more common than restraint or eating concern in this (non-clinical) sample.

The results of the current study should be interpreted in the context of its limitations. Firstly, the participants were female university students, identifying as Australian/Caucasian, with little variability in age. In addition, BMI was positively skewed in the sample, indicating a low representation of participants in the higher BMI range. Due to the lack of representation of various gender, socioeconomic, ethnic, and BMI groups within this study, the generalisability of the findings may be limited, and an examination of expanded sociocultural models of disordered eating in more diverse populations is warranted in future research. For example, Tylka [53] proposed an alternative conceptual model to explain body image and body change behaviours in boys and men, integrating additional sources of social influence (e.g., objectifying contexts, such as body building and certain gay communities) and forms of internalisation (e.g., internalisation of the mesomorphic ideal). Also, among the sample utilised for the current study, there were generally low rates of eating pathology reported, with low mean scores for some EDE-Q subscales, particularly restraint. It would be beneficial to replicate this study with populations displaying greater levels of eating pathology (e.g., clinical populations) to understand whether the current findings are generalisable to these groups. A further limitation of this study was the cross-sectional design, resulting in an inability to draw causal conclusions regarding the relationships among variables. Future longitudinal research could examine the causal pathways between thin-ideal internalisation, WBI, self-compassion, and eating disorder cognitions and behaviours to more effectively inform prevention and intervention programs. Future research may also consider mediated pathways from the sociocultural influences to disordered eating via self-compassion. For example, it is possible that WBI decreases self-compassion, which, in turn, increases eating disorder cognitions and behaviours. The belief that one is less attractive or less deserving due to their weight [42] is inconsistent with the self-compassionate stance of kindness and nonjudgement [27].

The results of the current study have implications for future research and practice related to the prevention and treatment of disordered eating. As discussed, the results support the inclusion of WBI alongside thin-ideal internalisation in current sociocultural models of disordered eating, providing evidence for the unique contribution of each construct to eating pathology. Further research is required to determine intersecting pathways between other constructs from thin-ideal and weight stigma models (e.g., weight stigmatising experiences to thin-ideal internalisation, or sociocultural pressures to be thin to WBI). The generalisability of extended models to more diverse and clinical samples must also be examined.

The expansion of sociocultural models of eating disorders, in turn, suggests an extension of current intervention strategies aimed at targeting the sociocultural factors associated with disordered eating. For example, interventions such as the Body Project, which utilise dissonance-based strategies to reduce thin-ideal internalisation, may be adapted to also target a reduction in WBI. It is likely that adapting this intervention to target both forms of internalisation will increase the efficacy of the intervention in reducing disordered eating. It is expected that the adaptation of interventions to address both thin-ideal internalisation and WBI may be particularly effective in increasing the efficacy of interventions for individuals with higher BMI, given the significant correlation between BMI and WBI [20,22], which was also observed in the current study. Further research may also investigate the impact of interventions that address both forms of internalisation across BMI categories.

## 5. Conclusions

In conclusion, the current study highlights the complementary nature of thin-ideal internalisation and weight bias internalisation by demonstrating that including both sociocultural constructs in predictive models provides a more comprehensive understanding of eating disorder cognitions and behaviours. In addition, the findings suggest that self-compassion may buffer the effects of thin-ideal internalisation on restraint and weight bias internalisation on eating concern. Future research should expand sociocultural models of body image and eating disturbance to include both forms of internalisation to improve theoretical understanding and predictive accuracy and inform future interventions.

## Figures and Tables

**Table 1:** Descriptive statistics and correlations.

		Mean	SD	α	1	2	3	4	5	6	7	8
1	Age	31.48	10.62	-								
2	BMI	26.66	7.14	-	**0.24**							
3	Self-compassion	3.11	0.71	0.92	**0.21**	−0.09						
4	WBIS	3.95	1.51	0.94	**−0.13**	**0.41**	**−0.54**					
5	Thin-ideal Internalisation	3.22	0.92	0.82	**−0.28**	−0.05	**−0.35**	**0.54**				
6	Restraint	2.92	1.67	0.84	0.01	0.09	**−0.28**	**0.43**	**0.43**			
7	Eating Concern	1.50	0.44	0.83	**−0.21**	**0.13**	**−0.50**	**0.67**	**0.53**	**0.60**		
8	Shape Concern	4.04	1.71	0.91	**−0.10**	**0.30**	**−0.52**	**0.81**	**0.57**	**0.59**	**0.78**	

Notes. *N* = 475; α = Cronbach’s alpha reliability. Significant correlations are shown in **bold**; |*r*| ≥ 0.10 are significant at *p* < 0.05; |*r*| ≥ 0.13 are significant at *p* < 0.01; |*r*| ≥ 0.24 are significant at *p* < 0.001.

**Table 2:** Hierarchical regression of thin-ideal internalisation, WBIS, self-compassion, ‘thin-ideal internalisation × self-compassion’, and ‘WBIS × self-compassion’ on restraint.

Step	Variable	*R* ^2^	Adjusted *R*^2^	*R*^2^ Change	B	β	sr^2^
1	BMI	0.01	0.01	0.01	0.02	0.09	0.01
2	BMI	0.24	0.24	0.24	0.000	−0.00	0.00
	Self-compassion				−0.12	−0.05	0.00
	Thin-ideal internalisation				0.50 ***	0.28 ***	0.05 ***
	WBI				0.28 ***	0.25 ***	0.03 ***
3	BMI	0.25	0.24	0.01	0.00	0.01	0.00
	Self-compassion				−0.14	−0.06	0.00
	Thin-ideal internalisation				0.50 ***	0.28 ***	0.05 ***
	WBI				0.27 ***	0.24 ***	0.02 ***
	Thin-ideal internalisation × self-compassion				−0.27 *	−0.11 *	0.01 *
	WBI × self-compassion				0.040	0.028	0.001

Notes. * *p* < 0.05, *** *p* < 0.001.

**Table 3:** Hierarchical regression of thin-ideal internalisation, WBI, self-compassion, ‘thin-ideal internalisation × self-compassion’, and ‘WBIS × self-compassion’ on eating concern.

Step	Variables	*R* ^2^	Adjusted *R*^2^	*R*^2^ Change	B	β	sr^2^
1	BMI	0.08	0.07	0.08	0.01 ***	0.19 ***	0.03 ***
	Age				−0.01 ***	−0.25 ***	0.06 ***
2	BMI	0.51	0.51	0.44	−0.005	−0.08	0.00
	Age				−0.00	−0.04	0.00
	Self-compassion				−0.10 ***	−0.16 ***	0.02 ***
	Thin-ideal internalisation				0.09 ***	0.19 ***	0.02 ***
	WBI				0.15 ***	0.51 ***	0.11 ***
3	BMI	0.53	0.52	0.01	−0.01	−0.08	0.00
	Age				−0.00	−0.03	0.00
	Self-compassion				−0.11 ***	−0.18 ***	0.02 ***
	Thin-ideal internalisation				0.09 ***	0.18 ***	0.02 ***
	WBI				0.15 ***	0.50 ***	0.10 ***
	Thin-ideal internalisation × self-compassion				0.00	0.00	0.00
	WBI × self-compassion				−0.04 **	−0.11 **	0.01 **

Notes. ** *p* < 0.01, *** *p* < 0.001.

**Table 4:** Hierarchical regression of WBIS, thin-ideal internalisation, self-compassion, ‘WBIS × self-compassion’, and ‘thin-ideal internalisation × self-compassion’ on shape concern.

Step	Variables	*R* ^2^	Adjusted *R*^2^	*R*^2^ Change	B	β	sr^2^
1	BMI	0.12	0.12	0.12	0.08 ***	0.34 ***	0.11 ***
	Age				−0.03 ***	−0.18 ***	0.03 ***
2	BMI	0.69	0.69	0.57	0.01	0.03	0.00
	Age				0.01 *	0.06 *	0.00 *
	Self-compassion				−0.27 ***	−0.11 ***	0.01 ***
	Thin-ideal internalisation				0.41 ***	0.22 ***	0.03 ***
	WBI				0.70 ***	0.62 ***	0.16 ***
3	BMI	0.69	0.68	0.00	0.01	0.03	0.00
	Age				0.01 *	0.06 *	0.00 *
	Self-compassion				−0.27 ***	−0.11 ***	0.01 ***
	Thin-ideal internalisation				0.41 ***	0.22 ***	0.03 ***
	WBI				0.70 ***	0.62 ***	0.16 ***
	Thin-ideal internalisation × self-compassion				0.05	0.02	0.00
	WBI × self-compassion				−0.02	−0.02	0.00

Notes. * *p* < 0.05, *** *p* < 0.001.

## Data Availability

The data presented in this study are available on request from the corresponding author because of the conditions of ethical approval.

## Abbreviations

The following abbreviations are used in this manuscript:

BMI	Body mass index
WBI	Weight bias internalisation
